# Organizational Resources in Rare Cancer Outcomes: Survival Analysis After Surgery for Pheochromocytoma and Paraganglioma

**DOI:** 10.3390/cancers17233884

**Published:** 2025-12-04

**Authors:** Kelvin Memeh, Sara Abou Azar, Nicholas R. Suss, Tanaz M. Vaghaiwalla

**Affiliations:** 1 Division of Surgical Oncology, Department of Surgery, The Ohio State University Comprehensive Cancer Center—The James, Columbus, OH 43210, USA; 2 Department of Surgical Oncology, Roswell Park Comprehensive Cancer Center, Buffalo, NY 14203, USA; 3 Department of Surgery, University of Chicago Medicine, Chicago, IL 60637, USA; 4 Division of Endocrine Surgery, Department of Surgery, University of Miami Miller School of Medicine, Miami, FL 33136, USA

**Keywords:** pheochromocytoma, paraganglioma, malignant, volume, surgical resection, survival

## Abstract

**Background**: For very-rare cancers such as pheochromocytoma and paraganglioma (PPGL), center-level case volume is uniformly low, rendering the traditional volume–outcome paradigm uninformative. This study examines whether cancer programs’ institutional resources, after adjusting for tumor-specific case volume, impact overall survival (OS) after surgery. **Methods**: The 2004–2021 National Cancer Database was queried for patients with a diagnosis of PPGL with malignant potential. Demographics, clinicopathologic characteristics, socioeconomic status, and treatment and survival variables—together with program resource tier (high resource = Academic/Research + Comprehensive Community Cancer Programs; low resource = Community Cancer + Integrated Network Programs), were extracted. IPW-Cox proportional hazard model and survival analysis were performed. **Results**: 1306 patients were identified, of whom 1066 (81.6%) were treated at high-resource programs. Mean age was 59.0 years and 55.1% were female (*n* = 719). Median follow-up was 61.7 months (maximum 207 months). Mortality was 28.3% (*n* = 278). Age, race, median income, tumor size, and surgical approach did not differ by resource tier. Patients treated at high- vs. low-resource programs differed by Charlson– Deyo score (*p* = 0.008), gender (*p* = 0.033), insurance status (*p* = 0.004), and distance traveled to facility (*p* < 0.001). On adjusted survival analysis, treatment at a high-resource program was associated with improved OS (HR = 0.64, *p* = 0.043) and a mean survival advantage of 23 months (*p* = 0.009) vs. a low-resource program. Age (HR = 1.03), tumor size >10 cm (HR = 4.18), and metastasis (HR = 4.17) independently predicted worse OS. **Conclusions**: Despite uniformly low PPGL case volumes nationally, treatment at high-resource cancer programs was associated with a 23-month longer mean survival and a 36% lower risk of death compared with low-resource cancer programs. Further studies are needed to identify the specific institutional factors that drive this survival advantage in rare cancers.

## Simple Summary

Pheochromocytomas and paragangliomas (PPGL) are very rare tumors, so most hospitals treat only a few cases. This study examines whether the level of institutional resources at cancer programs affects survival after surgery. Using the 2004 and 2021 National Cancer Database, 1306 patients were identified and included in the analysis. Most patients (82%) were treated at high-resource programs, including Academic/Research and Comprehensive Community Cancer Programs. After adjusting for patient and tumor characteristics, treatment at high-resource programs was associated with meaningful and statistically significant improvement in overall survival—a 23-month longer mean survival or a 36% lower risk of death—compared with low-resource programs. Older age, larger tumors, and metastasis were linked to worse outcomes. These findings suggest that even when case volumes are low, institutional resources may influence survival in PPGL, highlighting the need to identify specific program-level factors that drive this benefit.

## 1. Introduction

Pheochromocytomas and paragangliomas (PPGL) are rare complex tumors arising from chromaffin cells of the adrenal medulla or extra-adrenal sympathetic ganglia [1,2]. Due to their indolent nature, most PPGLs are diagnosed incidentally, with 15% to 25% of patients subsequently developing distant metastasis—driving five-year overall survival as low as 50% in advanced cases [3,4,5,6]. The management of PPGL is uniquely challenging: pre-operative optimization requires coordinated specialized endocrinology, anesthesia, and surgical and pathologist input, while the need for lifelong surveillance further complicates care [7]. Such treatment complexity naturally raises the question of where these patients should be treated, as cumulative institutional experience with tumors might influence outcomes.

In many common malignancies, hospitals with higher procedure volumes consistently outperform their low-volume peers. Specifically, several volume–outcome studies spanning breast, thyroid, head and neck, pancreatic, and colon cancer domains have linked treatment in high-volume centers to superior overall survival, thus supporting arguments for policies that regionalize complex surgery and set minimum-volume thresholds [8,9,10,11,12,13,14,15,16]. But rare cancers, like PPGL, challenge this paradigm. With an incidence below one case per 100,000 person-years, most cancer centers treat too few patients to achieve the procedural expertise conferred by PPGL-specific volume [2,17]. That is, for PPGL, a traditional “high-volume versus low-volume” framework cannot meaningfully describe levels of expertise across programs. Measurable criteria are needed to identify cancer programs that achieve superior cancer-specific outcomes despite low overall case volume.

Organizational learning and absorptive capacity theory suggests that highly absorptive hospitals may compensate for scarcity of tumor-specific experience by leveraging institutional knowledge and resources, including specialized clinical staff, multidisciplinary protocols, and quality improvement infrastructure, built through experience with more prevalent cancers [18,19,20,21]. Academic and Comprehensive Community Cancer Programs (ACAD/CCCP) are explicitly accredited for such resources, whereas Community Cancer and Integrated Network Programs (CCP/INCP) typically have more limited infrastructure [22,23]. Whether these high-resource cancer programs (ACAD/CCCP) translate their broader capacity into survival benefits for patients with ultra-rare tumors remains an empirical question of both clinical and policy relevance.

The National Cancer Database (2004–2021) was utilized to examine whether surgery for PPGL at high-resource cancer programs (ACAD/CCCP) is associated with improved overall survival compared with low-resource programs (CCP/INCP). The analysis further tested whether any observed advantage persists after accounting for the small variation in PPGL case volume that exists across programs. The underlying hypothesis was that institutional resources, rather than caseload, would yield a significant survival advantage in this rare endocrine malignancy.

## 2. Methods

### 2.1. Data Source and Patient Selection

This retrospective cohort study utilized data from the National Cancer Database (NCDB) [24] from 2004 to 2021. The NCDB aggregates de-identified patient information from over 1500 Commission on Cancer–accredited hospitals across the United States. Adult patients (≥18 years) diagnosed with PPGL were identified in the database using the International Classification of Disease for Oncology third edition (ICD-O-3) site codes C740, C741, and C749, and histology codes 8680 (Paraganglioma, Malignant), 8693 (Extra-adrenal Paraganglioma, Malignant), and 8700 (Pheochromocytoma). Only patients who underwent surgery were included in the analysis. Given the de-identified nature of the NCDB, this study is exempt from approval by an institutional review board.

### 2.2. Variable Selection

Cancer programs designated as Academic/Research or Comprehensive Cancer Centers, institutions typically expected to be highly resourced per the American College of Surgeons Commission on Cancer (CoC) accreditation standards [22,23], were classified as high-resource cancer programs (HRCPs). In contrast, Community and Integrated Cancer Network facilities were categorized as low-resource cancer programs (LRCPs) given their lower access to resources compared with HRCPs. For each facility, all PPGL resections across the study window were computed. For descriptive analysis, hospitals were classified into four prespecified strata: 1 case, 2–3 cases, 4–5 cases, and more than 5 cases over 18 years. Demographic variables (age, sex, and race), clinical characteristics (Charlson–Deyo comorbidity score, tumor size, and metastasis at diagnosis), treatment-related factors (surgical approach and receipt of chemotherapy), and socioeconomic indicators (median household income and insurance type) were retrieved. In addition, geographic characteristics, including residential area (metropolitan, urban, or rural) and distance from the treatment facility, were recorded.

### 2.3. Statistical Analysis and Modeling

Descriptive statistics were computed to characterize the study cohort. The distribution of the continuous variables was examined using histogram plots, and appropriate summary statistics were presented (median, with interquartile range). The categorical variables were summarized as frequencies (percentages). Differences in baseline characteristics between high-resource and low-resource cancer programs were assessed using the Wilcoxon rank-sum test for continuous variables and the Pearson chi-square test for categorical variables. Overall survival was estimated using the Kaplan–Meier method, and differences between facility volume groups were compared using the log-rank test.

To address selection bias by program type (HRCP versus LRCP), inverse-probability–weighted (IPW) survival models were utilized. We started by first generating inverse-probability weights to balance the assignment into treatment groups using a comprehensive set of pre-treatment covariates that capture socioeconomic variables, comorbidity, disease burden, and geography. This model adjusted for the likelihood of receiving care at either HRCP or LRCP. Using the generated weights, two complementary weighted survival models were then fit: (1) an IPW treatment effect survival model to estimate the adjusted difference in mean survival (in months), and (2) an IPW-Cox proportional hazards (CPH) model to estimate the adjusted hazard ratios (HRs).

Both survival models additionally adjusted for factors that are likely to impact survival, including demographic, clinical, and treatment-related factors. These models also accounted for within-facility correlation using clustered robust standard errors at the hospital level. Because nearly 99% of patients with unknown insurance status (70 out of 71 patients) were treated at HRCC, resulting in a highly skewed distribution, these patients were excluded to avoid introducing bias in the adjusted analyses. Furthermore, only patients with at least one year of survival data were included in the analysis. Further details of our statistical modeling are presented in the Supplementary Material.

Statistical significance was set at *p* < 0.05. All statistical analyses were performed using Stata 18 (StataCorp LLC, College Station, TX, USA) [25].

### 2.4. Sensitivity Analysis

To evaluate the robustness of the main findings, several checks were conducted. To exclude the impact of the skewed distribution of case volumes, we re-fitted the IP-weighted Cox model using case volume as a log-transformed continuous measure. Next, to evaluate the nature of any volume–outcome relationships within each resource tier, the IP-weighted Cox models were re-estimated separately, comparing the high-resource programs (Academic vs. Comprehensive Community) and the low-resource programs (Community vs. Integrated Network). Furthermore, the analysis tested whether the survival benefit associated with program resources varied by program case volume by introducing an interaction between a binary-level case-volume category and resource tier. A dummy variable was created to indicate a high-volume program if they resected 5 or more cases during the study period, and low-volume program otherwise. Finally, E-values for the resource-tier hazard ratios were calculated to quantify the minimum strength of an unmeasured confounder that would be required to negate the observed association between program resources and overall survival. Readers are referred to these resources for a review and further discussion of E-value methods for conducting sensitivity analyses for unmeasured confounding in observational study design [26,27,28].

## 3. Results

### 3.1. Baseline Characteristics

A total of 1306 adult patients with PPGL underwent surgical resection across 489 cancer programs between 2004 and 2021. Of these, 240 (18.4%) were treated at an LRCP, while 1066 (81.6%) received care at an HRCP. As summarized in Table 1, the groups were generally comparable in terms of vital status (approximately 28% deceased; *p* = 0.61), age at diagnosis (mean 59.0 ± 11.0 years; *p* = 0.89), and racial group composition (*p* = 0.80). However, a higher proportion of males (46.3% vs. 38.8%, *p* = 0.033) and patients with lower Charlson–Deyo comorbidity scores (*p* = 0.008) were treated in HRCP facilities. While other clinical parameters (tumor size, metastasis at diagnosis, surgical approach, and receipt of chemotherapy) did not differ significantly between the groups, days to surgery, disparities in health insurance status (*p* = 0.004), and distance from the treatment facility (*p* < 0.001) were noted (Table 1).

Of the 489 cancer programs in the NCDB dataset, 255 (52%) treated only a single PPGL case over the 18-year study period: 34.3% of Academic/Research Programs, 61% of Comprehensive Community Cancer Programs, 91% of Community Cancer Programs, and 55.2% of Integrated Network Cancer Program hospitals (Table 2). Even among Academic/Research programs, fewer than 20% managed >5 cases, underscoring a uniformly low PPGL-specific experience across the United States.

### 3.2. Unadjusted Survival Analysis

Univariable Cox regression modeling (Table S1—Supplementary Material) revealed that, not adjusting for other factors, the program resource tier (HRCP vs. LRCP) was not significantly associated with overall survival (HR 1.04, 95% CI 0.73–1.40; *p* = 0.937). In contrast, increasing age (HR 1.04, 95% CI 1.02–1.05; *p* < 0.001), tumor size >10 cm (HR 4.18, 95% CI 1.84–9.50; *p* = 0.001), the presence of metastasis at diagnosis (HR 4.76, 95% CI 2.62–8.65; *p* < 0.001), receipt of adjuvant chemotherapy (HR 3.96, 95% CI 2.45–6.41; *p* < 0.001), and a higher Charlson–Deyo comorbidity score (HR 1.21, 95% CI 1.05–1.39; *p* = 0.008) were each significantly associated with poorer survival outcomes (Table S1—Supplementary Material).

### 3.3. Adjusted Survival Analysis

After adjusting for potential confounders, including case volume at the hospital level, treatment at HRCP was associated with a mean survival advantage of approximately 23 months (95% CI 5.75–41.08 months; *p* = 0.009) compared to LRCP. Furthermore, the covariate distribution across program resource tier in the model was balanced, thus minimizing potential pre-treatment confounding. In addition, the overidentification test yielded non-significant results, confirming the adequacy of the model specification (*χ*^2^ = 13.3, *p* = 0.7158) (Table 3 and Table S2—Supplementary Material).

**Figure 1:**
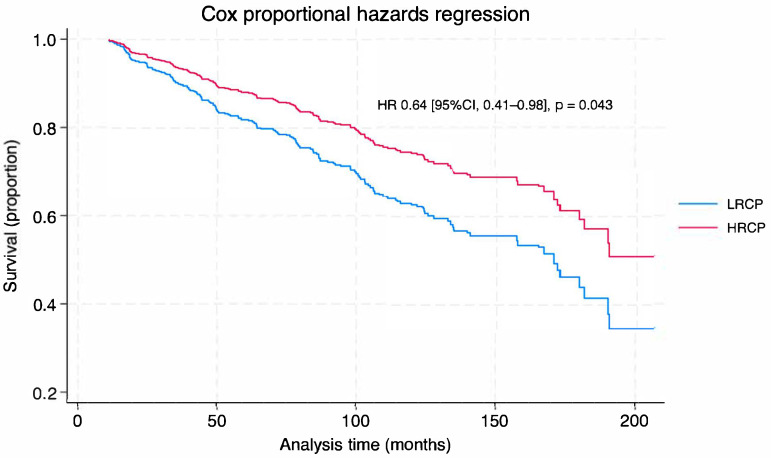
Cox Proportional Hazard Survival Curve by Facility Volume. LRCP: low-resource cancer program; HRCP: high-resource cancer program; Figure 1: Cox proportional hazard survival curve shows the probability of survival over time (months) in patients with pheochromocytoma/paraganglioma with malignant potential, surgically treated at low-resource cancer program (LRCP—blue line) vs. high-resource cancer program (HRCP—red line). Patients treated in HRCP had a 36% reduced risk of death compared to those treated in LRCP (*p* = 0.043).

In the IPW-Cox proportional hazards model, receiving treatment at an HRCP was associated with improved overall survival (HR 0.64 95% CI, 0.41–0.98; *p* = 0.043) (Figure 1, and Table 4). Importantly, after adjusting for all other factors, program case volume was not a predictor of overall survival. Instead, several other pre-treatment factors were observed to be independent predictors of overall survival: Female sex (HR 0.66, 95% CI 0.46–0.94) and having a private health insurance coverage (HR 0.31, 95% CI, 0.15–0.62) were associated with improved overall survival, whereas the presence of metastasis at diagnosis (HR 4.17, 95% CI, 1.23–14.14) and increasing age (HR 1.03, 95% CI, 1.01–1.05) were associated with a decrease in overall survival (Table 4). Lastly, the IPW-Cox model did not violate the proportional hazard assumptions (Schoenfeld residual test: *χ*^2^ = 21.50, *df* = 30, *p* = 0.6645).

### 3.4. Sensitivity Analysis

The robustness of the primary resource–tier finding was tested in four complementary models. First, to obviate any influence of arbitrarily defined caseload cut-points, the inverse-probability–weighted (IPW) Cox model was re-estimated with cumulative case volume entered as a log-transformed continuous covariate. The adjusted hazard ratio (HR) for treatment at a high-resource cancer program (HRCP) remained largely unchanged at 0.61 (95% CI, 0.39–0.96; *p* = 0.032), indicating that the survival advantage of HRCPs is insensitive to the functional form of the volume variable (Table S3—Supplementary Material).

Second, potential volume–outcome relationships within resource strata were explored. Among high-resource programs, Academic/Research hospitals exhibited a higher, but statistically insignificant, mortality risk relative to Comprehensive Community programs (HR, 1.65; 95% CI, 0.99–2.77; *p* = 0.057), a directionality likely reflecting referral of more advanced or medically complex PPGL cases to tertiary hospitals. Within low-resource programs, mortality did not differ between Integrated Network and Community Cancer Programs (HR, 0.84; 95% CI, 0.24–2.89; *p* = 0.779) (Table S3—Supplementary Material).

Third, effect-modification by case volume was assessed by introducing an interaction between resource tier and a binary volume indicator (<5 (low-volume) vs. ≥5 (high-volume) cumulative cases). Among low-volume hospitals, high-resource programs retained a significant survival advantage over low-resource programs (HR, 0.59; 95% CI, 0.35–0.99; *p* = 0.045). In high-volume settings, the point estimate continued to favor HRCPs (HR, 0.75; 95% CI, 0.33–1.69; *p* = 0.486)—though this was not statistically significant. The formal interaction term was non-significant (HR, 1.27; 95% CI, 0.48–3.34; *p* = 0.64), and volume itself was not associated with survival within either tier (HRs, 1.20 and 0.95 for HRCP and LRCP, respectively). These findings suggest that institutional resources, rather than caseload, drive the observed survival benefit. (Table S3—Supplementary Material).

Finally, an E-value of 2.50 (lower-bound E-value, 1.11) was estimated for the HRCP effect, signifying that an unmeasured confounder would need to be associated with both program resource tier (HRCP vs. LRCP) and mortality by a risk ratio of at least 2.5 to fully explain away the observed association in the study—an effect substantially stronger than any measured covariate in the analysis, except distant metastasis at diagnosis. (Table S3—Supplementary Material).

Collectively, these sensitivity checks demonstrate that the 36% reduction in mortality associated with HRCP care is robust to alternative caseload specifications, residual volume–outcome confounding, and unlikely to be nullified by plausible unmeasured factors.

## 4. Discussion

In this national cohort of 1306 patients who underwent resection for pheochromocytoma and paraganglioma (PPGL), care delivered in high-resource cancer programs (HRCPs), defined as academic and comprehensive community hospitals accredited for multidisciplinary capacity, was associated with a 36% reduction in mortality compared to low-resource programs (LRCPs), corresponding to a mean survival gain of almost two years. The observed survival advantage persisted when hospital case volume was modeled as a continuous logarithmic variable and did not vary by the modest differences in PPGL case volume that exist among U.S. cancer programs. Thus, when procedure-specific volume is intrinsically low, the breadth of organizational resources appears to be a principal determinant of long-term survival.

Decades of research in more common malignancies have linked high case volume to better outcomes, a finding that has encouraged regionalization policies and minimum-volume standards [8,9,10,11,12,13,14,15,16,29,30,31,32]. In these instances, the cancers are relatively common; thus, it is comprehensible that as a program’s exposure to a given cancer accumulates, so does its expertise, which should translate to better outcomes for patients treated for that specific cancer. However, in the instance of a rare cancer, like PPGL, even the busiest cancer programs struggle to accumulate reasonable case volume, with most having fewer than five resections across 18 years in the current analysis. This data suggest that, under such scarcity, program case volume becomes less significant, and robust organizational capabilities can substitute for case volume. HRCPs likely leverage specialized endocrine-anesthesia teams, protocolized catecholamine blockade, genetics teams, and quality-improvement infrastructure to transform sporadic PPGL encounters into reliable, high-quality care. While the analysis focused on PPGL, these findings may extend to other rare cancers where case volumes are intrinsically low, suggesting that organizational resources rather than procedural frequency could be critical for outcomes. Specific HRCP resources such as dedicated multidisciplinary tumor boards, specialized anesthesia and endocrine teams, protocolized perioperative management, and access to genetics and quality-improvement infrastructure likely contribute to the observed survival advantage and could serve as actionable targets for other rare cancer programs seeking to optimize patient care. In contrast, LRCPs lack the slack resources required for this type of organizational learning and knowledge translation that has been documented in the organizational learning research [19,20,21,33]. It is important to emphasize that these findings do not dismiss the importance of case volume; instead, they suggest that resources within a cancer program could amplify whatever experience can be gained and become critical for patient outcomes when sheer case numbers cannot accrue. Still, we observed that regardless of the cancer program volume (<5 (low-volume) vs. ≥5 (high-volume) cumulative cases), higher resource capacity continued to be associated with improved survival. Although we only found this to be statistically significant in the low-volume group, we must note that an HR of 0.75 observed in the high-volume group is arguably clinically significant. Potential reasons for a non-statistically significant result in this high-volume group might be related to the focus of this study on overall rather than disease-specific survival, the former of which can be influenced by the differences in baseline case mix and referral bias.

Lastly, using the E-value method, the extent to which a factor not accounted for in the model might affect the results was explored. An unmeasured factor not present in the model would need to be associated with both program resource tier and mortality by a risk ratio of at least 2.5, independent of measured covariates, to nullify the study findings entirely. For context, only a few factors in the cancer outcomes literature demonstrate such strong dual associations after adjusting for cancer stage, comorbidity, and other treatment-related factors. Reported mortality risk ratios for factors such as treatment delays (1.2–1.4), socioeconomic disparity measures (1.1–1.3), and the surgeon-volume effect are commonly well below the 2.5-fold threshold. Advanced cancer stage at diagnosis or severe comorbidity are typically the only factors shown to independently exceed the 2.5 effect threshold, both of which were accounted for in the model. Furthermore, other potential (and clinically relevant) unmeasured confounders—such as patient-level functional status (frailty)—were considered; although not directly captured in the NCDB dataset, such factors could influence referral patterns, treatment intensity, and mortality risks. However, much of this effect is likely already partially captured by the Charlson–Deyo comorbidity score in the model, making it unlikely that residual confounding from frailty would meet the 2.5 risk-ratio threshold associated with both resource tier and survival. Nonetheless, frailty and comorbid conditions are not perfect correlates.

The observations from this study carry several practical and policy implications. First, referral pathways should direct PPGL patients, particularly those who are older, have metastatic disease, or harbor large tumors, to HRCPs, regardless of historic PPGL case volume. Second, accrediting bodies might consider augmenting case volume thresholds with explicit resource-tier criteria, such as specialized critical care teams, formal genomic review, and virtual multidisciplinary tumor boards. Third, the finding that privately insured and geographically proximate patients were disproportionately treated in HRCPs underscores persistent inequities in access to specialized oncology care. Tele-oncology platforms, digital referral systems, and travel assistance programs initiated and maintained by HRCPs may help close these gaps.

## 5. Strengths and Limitations

This study has several strengths. By analyzing two decades of National Cancer Database data, it represents the most extensive U.S. series of surgically treated PPGL. Furthermore, it provides contemporary insights into outcomes for this ultra-rare cancer from a national cohort. The application of inverse-probability–weighted survival models enhanced causal inference by balancing measured confounders before estimating the effect of cancer program resources on survival. Finally, situating the findings within organizational-learning theory reframes the policy debate from a singular focus on case volume to a broader appreciation of organizational capacity, even after adjusting for the latter.

The study also has limitations. As with any retrospective registry analysis, residual confounding cannot be entirely excluded; key variables such as surgeon and oncologist-specific experience, intraoperative hemodynamics, recurrence patterns, and tumor stage and genetic profile are not captured in the NCDB. The absence of this information in the model could bias the study estimates. However, the calculated E-value of 2.50 is moderately strong, suggesting that only a confounder with a strong, unmeasured association with both treatment program and mortality could negate the observed effect in this study. Another limitation is the assumption that each center’s resource tier designated by the Commission on Cancer is homogeneous. In reality, variation likely exists within groups, as individual academic or comprehensive community programs may differ in unmeasured ways that might impact referral patterns and patient outcomes. We have attempted to account for this potential heterogeneity by clustering at the hospital level to capture inter-hospital variation. Importantly, CoC accreditation is a widely recognized and validated proxy for program resources and multidisciplinary capacity, supporting the use of this classification despite potential variability. Finally, we evaluated overall survival rather than disease-specific survival, as the NCDB does not capture cause of death that can be used to codify cancer-specific survival. Consequently, noncancer-related deaths may have been included in the analysis, potentially influencing the observed associations.

## 6. Conclusions

In summary, “where” PPGL patients are treated appears more consequential than “how many” similar cases a hospital has performed. When procedure-specific volume cannot accumulate, organizational resources, including the presence of specialized teams, standardized care pathways, and integrated quality improvement systems, distinguish high-performing cancer programs. Future work should identify the specific factors and processes that translate organizational capacity into survival gains and evaluate knowledge-transfer interventions that could allow lower-resource hospitals to approximate the outcomes achieved in HRCPs.

## Figures and Tables

**Table 1:** Baseline Characteristics of Patients by Cancer Program Resource Tier (2004–2021).

Characteristic	LRCP (*n* = 240)	HRCP (*n* = 1066)	Total (*n* = 1306)	*p*-Value
**Vital status**				0.61
Dead	44 (26.7%)	234 (28.6%)	278 (28.3%)	
Alive	121 (73.3%)	584 (71.4%)	705 (71.7%)	
**PPGL cases-count, median, IQR**	2 (1–5)	5 (2–11)	4 (2–10)	<0.001
**Case-count category**				<0.001
1 case	84 (35.0%)	171 (16.0%)	255 (19.5%)	
2–3 cases	71 (29.6%)	259 (24.3%)	330 (25.3%)	
4–5 cases	36 (15.0%)	162 (15.2%)	198 (15.2%)	
>5 cases	49 (20.4%)	474 (44.5%)	523 (40.0%)	
**Age at diagnosis, median, IQR**	58.5 (50.5–68.0)	58.0 (51.0–67.0)	58.0 (51.0–67.0)	0.9776
**Sex**				0.033
Male	93 (38.8%)	494 (46.3%)	587 (44.9%)	
Female	147 (61.3%)	572 (53.7%)	719 (55.1%)	
**Race/ethnicity**				0.80
Non-Hispanic White	173 (72.1%)	747 (70.1%)	920 (70.4%)	
Non-Hispanic Black	38 (15.8%)	196 (18.4%)	234 (17.9%)	
Hispanic	20 (8.3%)	77 (7.2%)	97 (7.4%)	
Asian/Pacific Islander	8 (3.3%)	37 (3.5%)	45 (3.4%)	
Other	1 (0.4%)	9 (0.8%)	10 (0.8%)	
**Charlson–Deyo comorbidity index**				0.008
0	154 (64.2%)	725 (68.0%)	879 (67.3%)	
1	48 (20.0%)	246 (23.1%)	294 (22.5%)	
2	23 (9.6%)	48 (4.5%)	71 (5.4%)	
≥3	15 (6.2%)	47 (4.4%)	62 (4.7%)	
**Days from diagnosis to first surgical procedure, mean ± SD**	0 (0–50)	12 (0–57)	6.5 (0–56)	0.015
**Tumor size**				0.60
<5 cm	74 (30.8%)	285 (26.8%)	359 (27.5%)	
5–10 cm	39 (16.2%)	181 (17.0%)	220 (16.9%)	
>10 cm	9 (3.8%)	50 (4.7%)	59 (4.5%)	
Unknown/not stated	118 (49.2%)	549 (51.5%)	667 (51.1%)	
**Metastatic status**				0.16
No	127 (53.1%)	515 (48.4%)	642 (49.2%)	
Yes	3 (1.3%)	33 (3.1%)	36 (2.8%)	
Unknown/missing	109 (45.6%)	517 (48.5%)	626 (48.0%)	
**Surgical approach**				0.64
Open	85 (37.1%)	399 (38.9%)	484 (38.6%)	
MIS	93 (40.6%)	382 (37.3%)	475 (37.9%)	
Unknown	51 (22.3%)	244 (23.8%)	295 (23.5%)	
**Receipt of chemotherapy**				0.32
No	230 (95.8%)	1024 (96.1%)	1254 (96.0%)	
Yes	5 (2.1%)	31 (2.9%)	36 (2.8%)	
Unknown	5 (2.1%)	11 (1.0%)	16 (1.2%)	
**Median income**				0.36
<$46,277	25 (13.1%)	154 (16.5%)	179 (15.9%)	
$46,277–57,856	44 (23.0%)	201 (21.5%)	245 (21.8%)	
$57,856–74,062	55 (28.8%)	223 (23.9%)	278 (24.7%)	
≥$74,063	67 (35.1%)	355 (38.0%)	422 (37.5%)	
**Insurance**				0.004
Uninsured	6 (2.5%)	37 (3.5%)	43 (3.3%)	
Private	120 (50.0%)	503 (47.2%)	623 (47.7%)	
Medicaid/other government	28 (11.7%)	111 (10.4%)	139 (10.6%)	
Medicare	85 (35.4%)	345 (32.4%)	430 (32.9%)	
Unknown	1 (0.4%)	70 (6.6%)	71 (5.4%)	
**Geographic location**				0.022
Metropolitan area	213 (91.8%)	851 (84.8%)	1064 (86.2%)	
Urban area	15 (6.5%)	122 (12.2%)	137 (11.1%)	
Rural area	4 (1.7%)	30 (3.0%)	34 (2.7%)	
**Travel distance**				<0.001
0–10 miles	86 (35.8%)	314 (29.5%)	400 (30.6%)	
10–25 miles	52 (21.7%)	249 (23.4%)	301 (23.0%)	
25–50 miles	31 (12.9%)	144 (13.5%)	175 (13.4%)	
50–100 miles	21 (8.8%)	124 (11.6%)	145 (11.1%)	
>100 miles	6 (2.5%)	113 (10.6%)	119 (9.1%)	
Unknown	44 (18.3%)	122 (11.4%)	166 (12.7%)	

Data are presented as median and interquartile range (IQR) for continuous variables and *n* (%) for categorical variables. Comparisons between low-resource cancer programs (LRCP) and high-resource cancer programs (HRCP) were made using the Wilcoxon rank-sum test or Pearson’s χ^2^ test as appropriate. Abbreviations: PPGL, pheochromocytoma and paraganglioma; SD, standard deviation; cm, centimeter; LRCP, low-resource cancer programs; HRCP, high-resource cancer programs; MIS, minimally invasive surgery.

**Table 2:** Case volume per program type across study period 2004 to 2021.

Facility Type (CoC Designation)	Case Count Distribution †				Total Hospitals
	1 Case	2–3 Cases	4–5 Cases	>5 Cases	
Academic/Research (ACAD)	61 (34.3%)	54 (30.3%)	28 (15.7%)	35 (19.7%)	178
Comprehensive Community (CCCP)	110 (60.8%)	57 (31.5%)	9 (4.9%)	5 (2.8%)	181
Community Cancer Program (CCP)	31 (91.2%)	3 (8.8%)	0 (0.0%)	0 (0.0%)	34
Integrated Network (INCP)	53 (55.2%)	28 (29.2%)	8 (8.3%)	7 (7.3%)	96
All facility types	255 (52.2%)	142 (29.2%)	45 (9.2%)	47 (9.6%)	489

† Percentages are calculated row-wise and may not sum exactly to 100.0% because of rounding. Abbreviations: CoC, Commission on Cancer; ACAD, Academic/Research Program; CCCP, Comprehensive Community Cancer Program; CCP, Community Cancer Program; INCP, Integrated Network Cancer Program.

**Table 3:** IPW—Adjusted Impact of Program Resources on Mean Overall Survival after Resection of PPGL.

Resource Tier	Overall Survival	95% CI	*p* Value
HRCP (months)	88.51	78.40–98.63	– –
LRCP (months)	65.10	51.24–78.95	– –
HRCP vs. LRCP			
Mean difference (months)	23.41	5.75–41.08	0.009
Relative difference (hazard ratio)	0.64	0.41–0.99	0.043

Omnibus overidentification test for covariate balance in the IPW treatment effect model: *χ*^2^ = 13.3, *p* = 0.7158; Schoenfeld residual test of proportional-hazards assumption in the IPW-Cox model, *χ*^2^ = 21.50, *df* = 30, *p* = 0.6645. Abbreviations: IPW, inverse-probability weights; PPGL, pheochromocytoma and paraganglioma; CI, confidence interval; HRCP, high-resource cancer programs; LRCP, low-resource cancer programs.

**Table 4:** IPW-Adjusted Cox Proportional Hazards Regression for Overall Survival.

Covariate	HR	95% CI	*p* Value
Institutional resource tier (HRCP vs. LRCP)	0.64	0.41–0.99	0.043
PPGL caseload (per additional case)	1.00	0.98–1.02	0.811
Age (per year)	1.03	1.01–1.05	0.004
Sex (female vs. male)	0.66	0.46–0.94	0.021
Race (vs. Non-Hispanic White)			
Non-Hispanic Black	0.98	0.64–1.49	0.926
Hispanic	0.46	0.20–1.07	0.071
Asian/Pacific Islander	0.16	0.03–1.04	0.055
Other	4.49	1.02–19.71	0.046
Charlson–Deyo comorbidity index (vs. 0)			
1	1.04	0.71–1.54	0.852
2	0.76	0.38–1.50	0.425
≥3	2.15	0.91–5.10	0.081
Metastasis at diagnosis (vs. no)			
Yes	4.17	1.23–14.14	0.022
Unknown/missing	1.74	0.98–3.08	0.060
Median income (quartile; vs. ≤$46,277)			
$46,277–57,856	1.37	0.79–2.36	0.263
$57,856–74,062	1.29	0.71–2.32	0.401
≥$74,063	1.25	0.74–2.12	0.399
Insurance (vs. uninsured)			
Private	0.31	0.15–0.62	0.001
Medicaid/other government	0.63	0.27–1.45	0.276
Medicare	0.52	0.24–1.12	0.096
Geographic location (vs. metropolitan)			
Urban area	1.16	0.59–2.28	0.677
Rural area	0.65	0.21–1.95	0.440
Travel distance (vs. <10 miles)			
10–25 miles	0.86	0.56–1.30	0.468
25–50 miles	0.77	0.43–1.38	0.373
50–100 miles	1.24	0.67–2.31	0.496
>100 miles	1.52	0.66–3.50	0.326

Model weighted by inverse-probability weights for resource tier; robust standard errors clustered on facility identifier (*n* = 687 patients; 34 deaths; 353 clusters). Reference categories shown in parentheses. Abbreviations: IPW, inverse-probability weights; HRCP, high-resource cancer programs; LRCP, low-resource cancer programs; PPGL, pheochromocytoma and paraganglioma.

## Data Availability

The National Cancer Database (NCDB) is a joint project of the Commission on Cancer (CoC) of the American College of Surgeons and the American Cancer Society. The de-identified data used in this study were provided by the CoC’s NCDB and the participating hospitals; neither the CoC nor the hospitals have verified or are responsible for the statistical validity of the analysis or the conclusions drawn by the authors.

## Supplementary Materials

The following supporting information can be downloaded at: https://www.mdpi.com/article/10.3390/cancers17233884/s1, Table S1. Unadjusted analysis: Factors impacting survival after adrenalectomy for pheochromocytoma; Table S2. Covariate balance before and after inverse-probability weighting; Table S3. Results of sensitivity analysis; Figure S1. Distribution of PPGL caseload per hospital.
