# Supplementary material for: Organizational Resources in Rare Cancer Outcomes: Survival Analysis After Surgery for Pheochromocytoma and Paraganglioma
Source: Cancers (Basel). 2025 Dec 4;17(23):3884. doi: 10.3390/cancers17233884 (PMC12691476; doi:10.3390/cancers17233884)
Supplement: Supplementary file 1 [file cancers-17-03884-s001.zip › cancers-3936458-supplementary.pdf]

Supplementary Materials

Supplementary Method Session

To address selection bias by program type (HRCP versus LRCP), we utilized inverse probability–weighted methods to fit two complementary survival models: an inverse probability–weighted treatment-effect survival model and an inverse probability–weighted Cox proportional hazards (CPH) model.

We started by first fitting the inverse-probability-weighted treatment-effect survival model. This model consists of two parts that are simultaneously fitted: a treatment-assignment model (which creates inverse-probability weights (IPWs) based on each patient's probability of receiving care at an HRCP versus an LRCP), and an outcome model (which utilizes the IPWs to estimate adjusted survival in months). In the treatment-assignment model, we included pre-treatment factors selected for their clinical relevance and established associations with the likelihood of receiving care at either HRCP or LRCP. These factors include disease burden (tumor size and metastasis at diagnosis), overall physical health (Charlson-Deyo Comorbidity Index), socioeconomic status (insurance status and annual median income), and geographical factors (distance from the treatment facility and residential area type – metro, urban, or rural). The outcome model incorporated all variables included in the treatment-assignment model and additional covariates likely to impact survival, including demographic variables (age, sex, and race), treatment-related factors (surgical approach and receipt of chemotherapy) and hospital case-volume (across 2004 – 2021). Case-volume was treated as an independent covariate, rather than in the propensity model, because its distribution overlapped poorly across facility types. This modelling decision preserved model stability while still accounting for institutional experience (i.e. case volume).

For the inverse-probability-weighted CPH model, the IPWs from the treatment-effect survival model were incorporated to estimate the hazard ratios for over survival – adjusting for the same covariates listed above including the case-volume. This modeling approach offers two key advantages: (1) it allows for transparent adjustment of pre-treatment confounders by balancing their distributions across the treatment groups – HRCC and LRCC – prior to estimating the survival outcome; and (2) it facilitates the estimation of adjusted survival differences in absolute (months) and relative (hazard ratios) terms. The survival models were fitted utilizing hospital-clustered robust standard errors to account for within-facility correlation. Because nearly 99% of patients with unknown insurance status (70 out of 71 patients) were treated at HRCC, resulting in a highly skewed distribution, these patients were excluded to avoid introducing bias in the adjusted analyses. Further, only patients with at least one year of survival data were included in the analysis. Statistical significance was set at $p < 0.05$. All statistical analyses were performed using Stata 18 (StataCorp LLC)

Table S1. Unadjusted Analysis: Factors Impacting Survival after Adrenalectomy for Pheochromocytoma.

Covariate	(Hazard Ratio)	95% CI	p-value
**LRCP vs. HRCP**	1.01	0.73 – 1.40	0.937
**Program Case count**	0.98	0.97 – 0.99	0.000
**Age**	1.04	1.02 – 1.05	0.000
**Sex**	0.80	0.62 – 1.01	0.058
**Race**			
Non-Hispanic Black	0.86	0.62 – 1.18	0.337
Hispanic	0.54	0.32 – 0.90	0.019
Asian, Pacific Islander	0.44	0.18 – 1.07	0.071
Other	1.59	0.46 – 5.54	0.463
**Co-morbidity**	1.21	1.05 – 1.39	0.008

**Tumor Size**			
5 – 10 cm	1.96	0.89 – 4.31	0.093
> 10cm	4.18	1.84 – 9.50	0.001
Unknown	2.34	1.22 – 4.46	0.010
**Time to Surgery**	1.00	1.00 – 1.00	0.360
**Surgical Approach**			
Minimally invasive	0.82	0.56 – 1.20	0.311
Unknown	0.30	0.12 – 0.74	0.009
**Positive Surgical Margin**	1.13	0.81 – 1.57	0.472
**Metastasis at Diagnosis**			
Yes	4.76	2.62 – 8.65	0.000
Unknown status	1.38	0.93 – 2.03	0.107
**Adjuvant Chemotherapy**			
Yes	3.96	2.45 – 6.41	0.000
Unknown status	1.34	0.61 – 2.90	0.456
**Health Insurance**			
Private	0.41	0.24 – 0.71	0.002
Medicaid/Uninsured	0.68	0.36 – 1.28	0.229
Medicare	0.86	0.50 – 1.48	0.584
Unknown	0.24	0.11 – 0.52	0.000
**Median Income**			
$46,277 – $57,855	1.02	0.69 – 1.50	0. 918
$57,856 - $74,063	0. 85	0.58 – 1.25	0. 418
> $74,063	0. 77	0.54 – 1.12	0.170
**Geographical location**			
Urban Area	1.15	0.79 – 1.67	0.474
Rural Area	0.95	0.43 – 2.08	0.899
**Distance from facility**	1.01	0.96 – 1.06	0.755

Table S2. Covariate Balance Before and After Inverse-Probability Weighting.

**Covariate**	**Std Diff (Raw)**	**Std Diff (Weighted)**	**Var Ratio (Raw)**	**Var Ratio (Weighted)**
**Metastasis at diagnosis (vs No)**				
Yes	0.1585	–0.1642	3.5397	0.4554
Unknown/missing	–0.1837	0.0801	1.1113	0.9689
**Charlson–Deyo comorbidity (vs 0)**				
1	0.0551	0.0342	1.0722	1.0484
2	–0.1053	0.0450	0.6712	1.2114
≥ 3	–0.0847	0.0520	0.6916	1.2942
**Insurance category (vs Uninsured)**				
Private	0.0060	0.1119	0.9934	1.0081
Medicaid/Other Gov.	–0.0423	–0.1287	0.9062	0.7762
Medicare	–0.0029	–0.0149	0.9911	0.9894

Covariate	Std Diff (Raw)	Std Diff (Weighted)	Var Ratio (Raw)	Var Ratio (Weighted)
**Median income (vs ≤ $46 277)**				
$46 277–57 856	–0.0283	–0.1164	0.9585	1.1899
$57 856–74 062	0.0306	–0.0322	1.0323	0.9632
≥ $74 063	–0.0196	–0.0589	0.9834	0.9726
**Travel distance (vs 0–10 miles)**				
10–25 miles	0.0225	0.0338	1.0178	1.0372
25–50 miles	–0.0622	0.0170	0.8854	1.0339
50–100 miles	0.1935	–0.0219	1.6712	0.9514
> 100 miles	0.2447	–0.1786	2.9918	0.6016
**Geographic location (vs Metro)**				
Urban area	0.1748	–0.0622	1.6588	0.8616

Std Diff = standardized difference; Var Ratio = variance ratio. Values of ∣Std Diff∣ < 0.10 and Var Ratio ≈ 1 indicate good balance.

Table S3. Results of Sensitivity Analysis.

	IPW - Cox Model (Adjusted Hazard Ratio)		
	**Effect**	**95%CI**	**P value**
Survival effect of resource tier using log of case volume	0.61	0.39 – 0.96	0.032
Survival effect differences within resource tiers			
ACAD vs. CCCP	1.65	0.99 – 2.77	0.057
INCP vs. CCP	0.84	0.24 – 2.89	0.779
Interaction of case volume and resource tier			
Resource effect in centers with < 5 cases (*i.e., HRCP vs. LRCP: when case volume < 5*)	0.59	0.35 – 0.99	0.045
Resource effect in centers with ≥ 5 cases (*i.e., HRCP vs. LRCP: when case volume ≥ 5*)	0.75†	0.33 – 1.69	0.486
Volume effect within HRCP (*i.e., High vs. low case volume in HRCP only*)	1.20†	0.47 – 3.34	0.635
Volume effect within LRCP (*i.e., High vs. low case volume in LRCP only*)	0.95	0.41 – 2.23	0.91

†Estimates obtained with lincom command after the IPW Cox model. The Interaction (HRCP × high-volume) HR = 1.27, 95 % CI 0.48 – 3.34, p = 0.64.

E- value

E-value point estimate = 2.50

E-value lower CI = 1.11

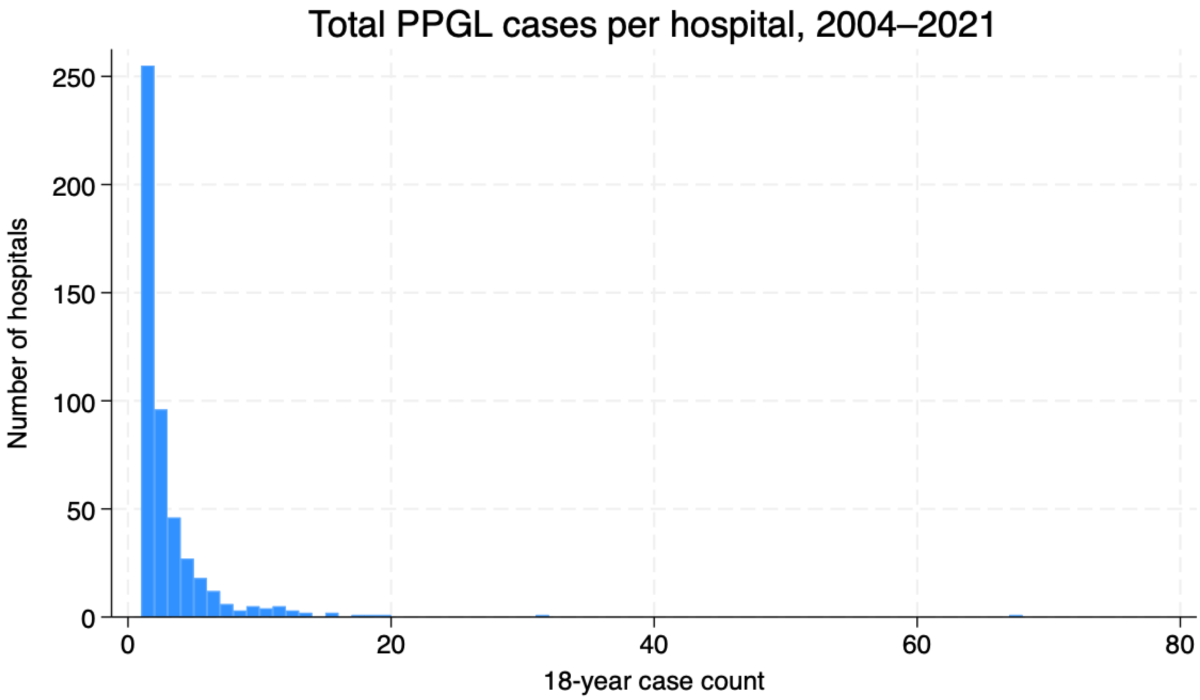

Figure S1. Distribution of PPGL caseload per hospital.
